# Utilization, Challenges, and Outlook of Cell of Origin Classification by Immunohistochemistry (IHC) in DLBCL

**DOI:** 10.3390/diagnostics16152378

**Published:** 2026-07-28

**Authors:** Anthony Hesser, Dorian Cohen, Gary Gustavsen, Barbara Furtado, Brad Thomas, Shady Gendy

**Affiliations:** 1 Health Advances LLC, Newton, MA 02466, USA; ahesser@healthadvances.com (A.H.); dcohen@healthadvances.com (D.C.); ggustavsen@healthadvances.com (G.G.); 2 US Medical Affairs, Hematology/Medical Diagnostics–Hematology, AstraZeneca, Gaithersburg, MD 20878, USA; barbara.furtado@astrazeneca.com; 3 Global Medical Affairs, Medical Diagnostics–Hematology, AstraZeneca, Gaithersburg, MD 20878, USA; brad.thomas1@astrazeneca.com

**Keywords:** diffuse large B-cell lymphoma, cell of origin, immunohistochemistry, gene expression profiling, biomarker, prognostic

## Abstract

**Background/Objectives:** Cell of origin (COO) classification in treatment-naïve (TN) diffuse large B-cell lymphoma (DLBCL) is universally recommended across guidelines as an essential prognostic tool, yet some patients are not tested or receive equivocal results that impede timely classification. **Methods:** The current study was informed by 18 qualitative, double-blinded phone interviews and a quantitative double-blinded survey of 60 hematologists/oncologists and 60 pathologists (including hematopathologists). Respondents were selected to represent academic and community practice settings based in the United States. **Results:** Hematologists/oncologists view COO as a valuable tool that provides prognostic insight and a more comprehensive diagnosis, with 83% of TN DLBCL patients tested for COO and 85% of hematologists/oncologists incorporating COO into treatment decisions. Hematologists/oncologists and pathologists prefer immunohistochemical (IHC) techniques over gene expression profiling (GEP) due to IHC’s accessibility, faster turnaround time, and relatively low cost. Although IHC workflows are routine, 10–15% of samples initially yield equivocal results. After repeat testing and/or multiple reviews and peer discussion, ~3% of submitted samples remain equivocal. Finally, we uncovered sentiment that the lack of pathology report standardization may undermine the use of COO classification in clinical decision-making. **Conclusions:** Our findings show that IHC is perceived as a reliable method for COO classification in TN DLBCL. Clinicians endorsed the need for guidelines to standardize pathology reports and establish definitive criteria for equivocal cases. These guidelines in combination with education to reinforce the clinical implications of COO can help mitigate testing barriers and increase the proportion of patients who receive timely COO classifications.

## 1. Introduction

Diffuse large B-cell lymphoma (DLBCL) is the most common subtype of non-Hodgkin lymphoma (NHL), accounting for approximately 30–40% of NHL cases [1]. DLBCL is an aggressive malignancy with a heterogeneous clinical course. Standard immunochemotherapy with the R-CHOP regimen can successfully cure ~60% of patients, but those with primary refractory or relapsed disease have a poor prognosis [2,3]. The inability to cure patients who progress motivated the investigation of tumor characteristics that can better stratify patients and guide treatment making decisions [4].

One stratification approach developed to understand the likelihood that patients progress on treatment is the cell of origin (COO) classification. Alizadeh et al. first performed gene expression profiling (GEP) with complementary DNA (cDNA) microarrays on fresh frozen (FF) tissue and found that DLBCL comprises at least two subtypes [1]. These subtypes have different molecular and prognostic features and correspond to the lymphoma’s cell of origin: a germinal center B-cell-like (GCB) subgroup, and an activated B-cell-like (ABC) subgroup [1]. As patients with GCB DLBCL (~60% of DLBCL cases in the US) tend to have better survival with standard therapies, COO has been recognized as an important prognostic biomarker in DLBCL and major international guidelines recommend reporting the COO classification in the DLBCL diagnostic work-up [2,5]. GEP approaches were further refined on formalin-fixed paraffin-embedded (FFPE) samples, which are more accessible in clinical practice than FF tissue [6].

Alternative approaches to COO classification using immunohistochemistry (IHC) were also developed, most commonly using three protein markers (CD10, BCL6, MUM1/IRF4) and the Hans algorithm. In the Hans algorithm, cases positive for CD10 (a germinal center marker) are classified as GCB; cases negative for CD10 are classified based on BCL6 and MUM1 expression: CD10(−)/BCL6(+)/MUM1(−) is deemed GCB, whereas CD10(−)/BCL6(−) or CD10(−)/BCL6(+)/MUM1(+) is classified as non-GCB [7]. Using this scheme, Hans et al. were able to classify patients into prognostic groups with outcomes comparable to the GEP-defined subtypes (76% 5-year overall survival in the IHC-defined GCB group vs 34% in the non-GCB group) [7]. Subsequent studies have generally supported the ability of IHC to identify patients likely to have poorer outcomes, including a recent study demonstrating the use of IHC to identify patients more likely to have a greater benefit from a newer therapy [8,9].

Although GEP assays are perceived to be the “gold standard” due to the more granular molecular information provided, GEP assays are more expensive and are not yet widely available in clinical practice due to requirements for specialized equipment and expertise in RNA workflows that are not common in hematopathology labs. In contrast, IHC is more broadly available and has a lower cost [7].

Despite the demonstrated clinical utility of COO and the availability of IHC-based approaches, limited published data exist in the US on COO testing rates, method preference, and challenges that may prevent determination of a COO classification. To understand the real-world use of COO and challenges associated with testing and reporting, we conducted a mixed-methods study that included double-blinded in-depth interviews and a qualitative survey of hematologists/oncologists, lab directors and pathologists (including hematopathologists). Clinicians from a mixture of academic and community centers were engaged to ensure results reflect the diverse practice settings in the United States.

## 2. Materials and Methods

### 2.1. Interviews

A series of 60-min double-blinded phone-based interviews were conducted with 8 hematologists/oncologists and 10 pathologists (including hematopathologists) from both academic (28%) and community practice (72%) settings in the United States. These discussions provided qualitative insight into current practices and challenges and informed design of a quantitative survey.

### 2.2. Survey Methodology

A double-blinded, online quantitative survey was conducted with United States-based hematologists/oncologists (*n* = 60) and lab directors and pathologists (including hematopathologists) (*n* = 60) from September to October 2025. To ensure methodological quality and integrity, survey respondents were recruited by a third party in compliance with industry standards, supplemented with additional outreach to a proprietary database. Ethical review and approval were waived for this study by Advarra CIRBI Platform, using the Department of Health and Human Services regulations found at 45 CFR 46.104(d)(2), and the IRB determined that the research project is exempt from IRB oversight.

Hematologist/oncologist survey respondents were required to be board-certified in medical or hematological oncology, dedicating greater than 50% of their time to direct patient care, and see on average 10 or more patients with DLBCL per year. Lab directors and pathologist survey respondents were required to be board-certified in at least one American Board of Pathology subspecialty, performing anatomic pathology (e.g., histology, cytology, flow cytometry, immunohistochemistry) in their primary laboratory, and processing on average 2 or more DLBCL samples per month. Lab directors were required to either have personal involvement in the development/supervision of laboratory workflows or supervision of pathologists. Pathologists were required to either have personal involvement in conducting tests or reviewing and releasing test results.

To ensure diversity of survey respondents, additional screening questions were used to collect information on the respondent’s training and primary practice setting. Participants that met the criteria above were invited to complete a 20-min online questionnaire. The hematologist/oncologist respondent demographics are displayed in Table 1 and lab director/pathologist respondent demographics are displayed in Table 2.

The survey sought to evaluate the current COO testing landscape by examining testing patterns in COO classification among DLBCL patients in the US, utilization of COO classification in clinical practice, and current challenges with testing using a combination of multiple choice, forced rank, and rating questions. The survey was designed to accommodate respondents across a variety of practice settings, patient volumes, hematopathology expertise, and geographical regions. To ensure consistency of responses, the following definitions were provided at the start of the survey:

*Cell of origin testing refers to the determination of GCB (germinal center B-cell) or non-GCB, including ABC (activated B-cell) or unclassified DLBCL*.

*Reflex protocol refers to a secondary/follow-up laboratory test that is performed automatically based on the results of an initial test. A reflexed test would not require a separate order*.

## 3. Results

### 3.1. Perceived Utility of COO

**Figure 1:**
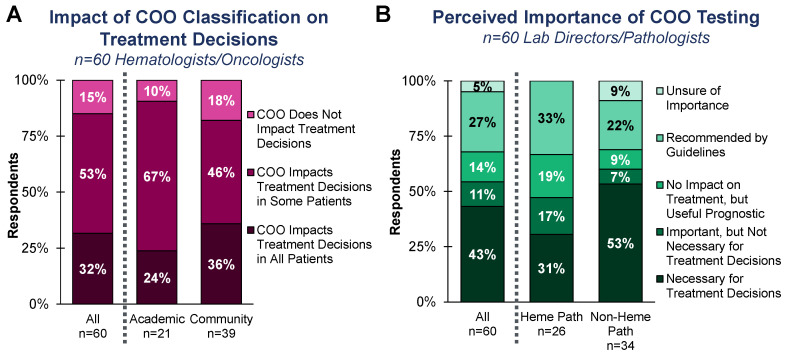
Use of COO classification in treatment decisions. (**A**) Hematologist/oncologist use of COO classification in treatment decisions. (**B**) Lab director and pathologist perception of why COO testing is important for clinical decisions. Percentages are rounded to the nearest whole number; therefore, totals may not equal 100% because of rounding.

Hematologist/oncologist interviews uncovered sentiment that COO classification provides a more complete diagnosis for DLBCL and that the COO classification may guide therapy selection. Of the US hematologists/oncologists surveyed, 53% use the COO classification to inform treatment selection for some patients (Figure 1A). An additional 32% of hematologists/oncologists report using COO classification as a factor in treatment decisions for all their patients. Pathologists (including hematopathologists) consider COO classification to be important, but only 54% of surveyed lab directors/pathologists (including hematopathologists) believe that hematologists/oncologists consider COO classification in treatment decisions (Figure 1B).

### 3.2. Current COO Testing Rate

**Figure 2:**
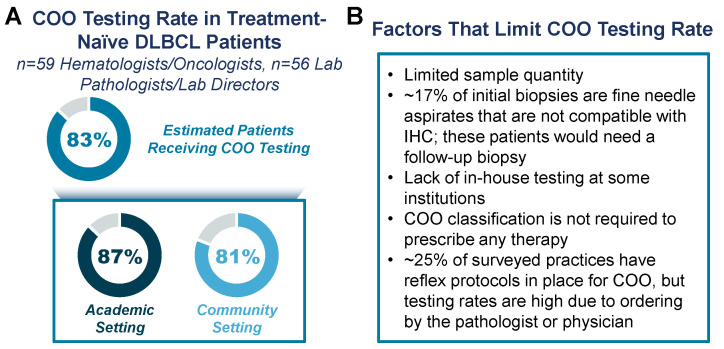
COO testing rate in TN DLBCL. (**A**) Excludes one hematologist/oncologist who reported that their patients do not receive COO testing and excludes lab directors/pathologists (including hematopathologists) who work at commercial/reference laboratories. (**B**) Rationale for why some patients never receive COO testing mentioned in hematologist/oncologist and pathologist interviews.

COO classification may be ordered by the managing physician, ordered by the pathologist at initial lymphoma/DLBCL diagnosis, or performed as a reflex test for any DLBCL diagnosis. While COO classification is universally recommended by guidelines, real-world testing rates from the survey were 87% for respondents at academic settings and 81% for those in community settings (Figure 2A). Based on the survey results, we estimate that 83% of DLBCL patients in the US are tested for COO (Figure 2A), assuming that ~58% of DLBCL patients in the US are managed in community settings [10].

Hematologists/oncologists and pathologists (including hematopathologists) cited several factors in our interviews that could explain why some patients are not tested for COO (Figure 2B). First, specimen quantity may be limited. Although COO is a priority, other tests, including flow cytometry, IHC panels for T- and B-cell markers, and double-/triple-hit fluorescence in situ hybridization (FISH) testing, also compete for limited tissue. Second, pathologists (including hematopathologists) estimated that 17% of samples received for DLBCL workup are in the form of a fine-needle aspirate (FNA), which is viewed as insufficient for architectural assessment needed to reliably establish a diagnosis. These patients would require a core needle, incisional, or excisional biopsy for further testing. Third, some community settings lack validated tests in-house for one or more of the Hans algorithm markers. Sending out these tests incurs a longer turnaround time. Fourth, because COO classification is not currently required to prescribe any therapy, the hematologist/oncologist or pathologist may choose to forgo testing, particularly for patients for whom physicians believe that the choice of therapy is clear. Finally, because only 25% of US practices have reflex protocols in place, miscommunication between hematologist/oncologist and pathologist could result in COO never being ordered for a patient. However, pathologists (including hematopathologists) think this situation is rare as COO is well-established in pathology testing algorithms and would be ordered regardless of any reflex protocol.

### 3.3. COO Classification Techniques and Concordance

**Figure 3:**
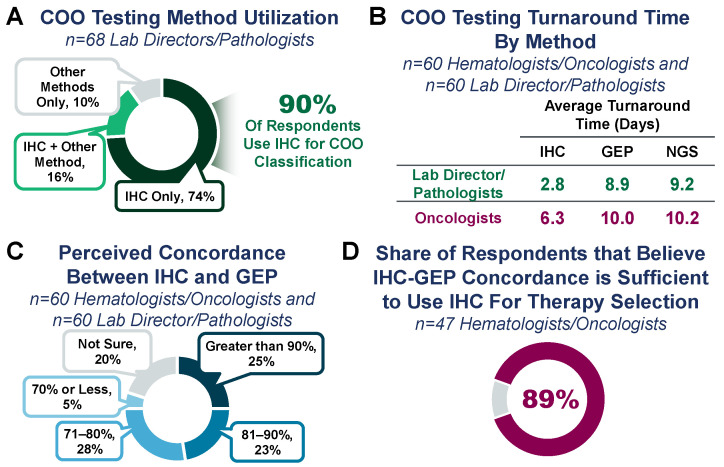
COO classification techniques. (**A**) Distribution of methods used for COO classification. Other methods include GEP- and NGS-based methods. Includes all ten interviewed pathologists and 58 surveyed lab directors/pathologists (including hematopathologists). Two surveyed lab directors who were not sure which method(s) their lab uses were excluded. (**B**) Estimated time to return a COO classification after testing. (**C**) Perceived concordance of COO classification between IHC and GEP approaches. (**D**) Share of hematologists/oncologists that are comfortable making treatment decisions based on COO classifications determined by IHC. Excludes hematologists/oncologists who answered “I am not sure” for a concordance estimate question. Percentages are rounded to the nearest whole number; therefore, totals may not equal 100% because of rounding.

The Hans algorithm using IHC techniques is by far the most established approach, with 90% of practices surveyed in the US using IHC (Figure 3A). The preference for IHC is due to its short turnaround time (typically less than 3 days once received by pathologist), broad availability, relatively low cost, and generally favorable insurance coverage and reimbursement (Figure 3B). Note that pathologists generally report turnaround times 3–4 days longer than hematologists/oncologists, reflecting omission (pathologists) versus inclusion (hematologists/oncologists) of specimen transport and accessioning time. Turnaround times are generally low because IHC is widely available, allowing for testing to be performed within the patient’s health system (71% of surveyed hematologists/oncologists have access to IHC testing in-house or in the same health system).

Of the surveyed lab director/pathologists (including hematopathologists), 16% report using other COO classification methods (e.g., GEP and NGS) with IHC to validate IHC results for some patients or to provide more granular DLBCL subtyping for challenging cases. In these situations, IHC is usually performed first to provide an initial COO classification. Compared to IHC, both GEP and NGS techniques are more costly to patients, slower to return results (4–6 days longer turnaround time than IHC), and often require send-out to specialized labs that can be challenging to identify (Figure 3B). Despite growing interest in GEP and NGS, hematologists/oncologists and pathologists (including hematopathologists) expect IHC to continue to be the dominant technique used over the next several years.

Most hematologists/oncologists and lab directors/pathologists (including hematopathologists) perceive the concordance between IHC and GEP to be 70–90%, consistent with published estimates (Figure 3C) [11,12]. Of the 47 hematologists/oncologists that were comfortable estimating the IHC-GEP concordance, 89% are comfortable using COO classifications determined via IHC for treatment selection (Figure 3D).

### 3.4. COO Classification Challenges

**Figure 4:**
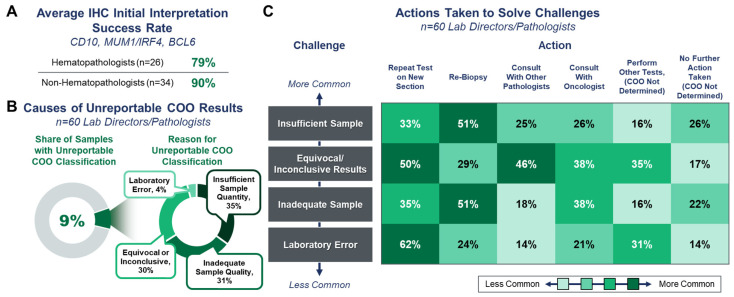
Challenges with unreportable COO classifications. (**A**) Average initial interpretation success rates using IHC refers to the share of samples that have a COO classification assigned without requiring a second opinion or repeated test. (**B**) Share of samples that underwent COO testing, but a COO classification could not be assigned due to insufficient sample quantity, inadequate sample quality, equivocal test results, or lab error. (**C**) Actions taken by pathologists (including hematopathologists) to solve challenges that may result in an inability to assign a COO classification.

Although IHC is an established and routine technique for COO classification, it has not been standardized, with pathologists (including hematopathologists) citing limited available guidance on scoring and interpretation. Pathologists in our survey estimated that 10–15% of samples initially yield equivocal results, which could arise from any of the three Hans algorithm antibodies. Pathologists report that interpreting weakly positive CD10 staining and focal MUM1/IRF4 staining are the most challenging scenarios. Notably, board-certified hematopathologists reported a lower initial interpretation success rate of 79%, compared to 90% reported by non-hematopathology certified pathologists (Figure 4A).

Most equivocal results are resolved through peer review with another pathologist, usually within the same laboratory. Smaller pathology groups, especially those without hematopathology expertise, are more likely to send samples to an external laboratory for a second opinion. After second opinions, an estimated ~3% of submitted specimens remain equivocal (Figure 4B). To reduce this rate further, pathologists (including hematopathologists) would welcome standardization of staining protocols, scoring, and interpretation. Improvements in antibody specificity would also be welcome.

For ~6% of submitted samples, COO cannot be classified due to insufficient sample to perform all required tests or because the quality of the sample is inadequate (e.g., highly necrotic) (Figure 4B). Although repeat biopsies pose a significant patient burden, ~50% of pathologists (including hematopathologists) may request a repeat biopsy for patients who have insufficient initial biopsies to perform COO classification and other tests (Figure 4C). Similarly, 60% of hematologists/oncologists would recommend a repeat biopsy for patients who do not receive a COO classification.

### 3.5. COO Reporting

**Figure 5:**
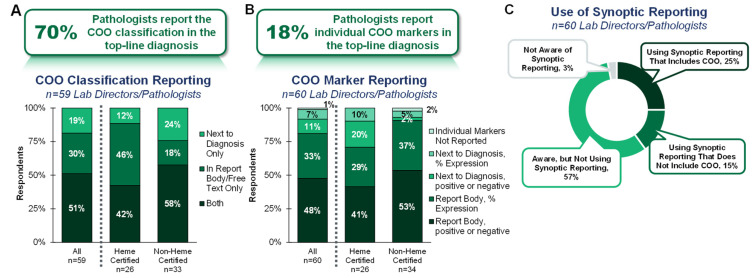
Pathology report variation. (**A**) COO classification may be reported next to DLBCL diagnosis (topline), in the body or free text section of the report, or both. One pathologist only reports individual markers and does not assign a COO classification was excluded. (**B**) Similarly, individual markers may be reported next to the DLBCL diagnosis, in the body or free text section of the report, or both. (**C**) Familiarity and use of synoptic reporting for DLBCL. Percentages are rounded to the nearest whole number; therefore, totals may not equal 100% because of rounding.

COO classifications determined by IHC are typically reported to the ordering physician at the same time as the DLBCL diagnosis rather than in an addendum following the initial diagnosis. The structure and contents of the pathology report are highly variable, not just between institutions but sometimes between pathologists at the same institution. Of the surveyed practices, 70% include the COO classification in the top-line diagnosis of the report (i.e., the final reported diagnosis), with the remaining 30% only including the COO classification in the detailed body of the report (Figure 5A). The interpretation of each marker is usually reserved for the body of the report, but 18% of practices include these results in the top-line diagnosis (Figure 5B).

Of all the surveyed lab directors/pathologists (including hematopathologists) who use IHC for COO classification, 43% claim that ABC is one of the COO classifications that they report, which is a classification that cannot be determined from IHC alone. This finding was independent of certification, with 41% of those with hematopathology certification and 44% of those without hematopathology certification using ABC and non-GCB interchangeably.

**Figure 6:**
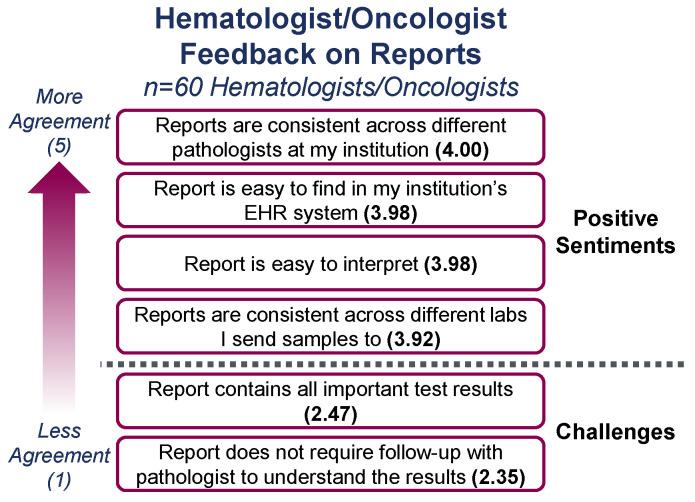
Hematologist/oncologist feedback on pathology reports including COO. Average level of agreement with statements around COO reporting, where 5 indicates strong agreement and 1 indicates strong disagreement.

While many hematologists/oncologists noted good readability and consistency, non-standardized pathology reports still left over 50% of hematologists/oncologists citing interpretation difficulties or the need to follow up for missing data (Figure 6). Greater multidisciplinary team (MDT) engagement may alleviate some challenges, yet only 25% of the laboratory directors/pathologists (including hematopathologists) surveyed report that MDTs are always engaged, with logistics and added workload as major barriers.

**Figure 7:**
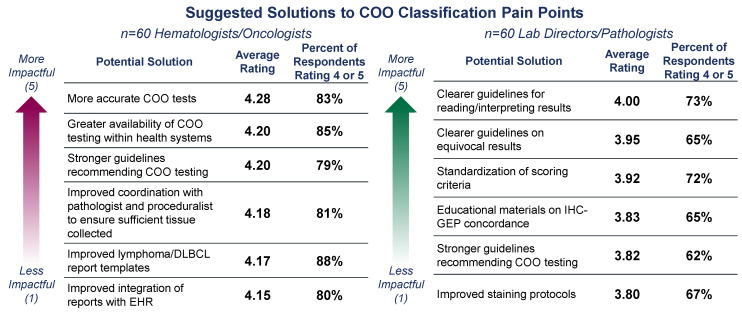
Suggested solutions to challenges. Average ratings are shown, where 5 indicates that the solution would be impactful and 1 indicates the solution would not be impactful. Only the top ranked solutions from a longer list are shown.

Another potential solution to promote standardization is to improve report templates (Figure 7), potentially adopting a synoptic reporting style where discrete data fields are used over prose and free text. Unlike other cancers such as breast, lung and colorectal cancer where synoptic reporting is the standard, only 25% of surveyed pathologists (including hematopathologists) use a synoptic reporting template that includes the COO classification (Figure 5C). Some interviewed pathologists (including hematopathologists) believe the existing DLBCL templates lack key markers and tend to be less practical.

## 4. Discussion

COO classification in TN DLBCL is recommended by international guidelines and supported by substantial evidence as a prognostic factor. Our findings show high perceived value: 85% of surveyed hematologists/oncologists report using COO classification in treatment decisions (for some or all patients), and 54% of surveyed laboratory directors/pathologists (including hematopathologists) believe that hematologists/oncologists use COO in treatment decisions (Figure 1A,B). Despite high use in clinical decision-making, most hematologists/oncologists and pathologists (including hematopathologists) requested stronger guidelines recommending COO testing (Figure 7). Raising awareness among pathologists that COO may inform treatment selection could reinforce the importance of performing COO testing and clearly reporting results, especially as hematologists/oncologists expect to see therapies that incorporate COO-based stratification.

Drawing on a diverse US cohort across regions and practice types, our survey suggests a real-world COO testing rate of ~83% in TN DLBCL, with slightly higher testing at academic centers (87%) than in the community (81%) (Figure 2). The primary barrier to COO classification is specimen adequacy, both quantity and quality, amid competing testing priorities for limited tissue. Improvements suggested by hematologists/oncologists and pathologists (including hematopathologists) include reinforcing MDT collaboration across hematology/oncology, pathology, interventional radiology, surgery, and other care-team members; emphasizing sample collection guidelines to reduce repeat biopsies; and recognizing that FNAs (~17% of cases) are often insufficient for a complete lymphoma workup, while acknowledging the complexity and challenges of excisional and incisional biopsies in some circumstances. MDT discussions can help coordinate the most appropriate approach, with 81% of hematologists/oncologists endorsing better coordination with the pathologist and proceduralist to ensure adequate tissue (Figure 7). Finally, implementing reflex protocols that include guideline-recommended risk stratification, including COO classification for TN DLBCL, can mitigate miscommunication between hematologists/oncologists and pathologists.

Of the surveyed pathologists (including hematopathologists), 90% use IHC for COO classification due to its short turnaround time (typically less than 3 days once received by pathologist), broad availability, relatively low cost to patients, and generally favorable coverage and reimbursement (Figure 3A). While hematologists/oncologists acknowledge that COO testing with GEP or NGS approaches can provide more granular patient stratification, these approaches are more costly than IHC, slower to return results (typically 4–6 days longer turnaround time than IHC), and often require a send-out to specialized labs with RNA workflows, which can be challenging to identify. Despite growing interest in GEP and NGS as more targeted DLBCL therapies are developed, hematologists/oncologists and pathologists (including hematopathologists) expect IHC to continue to be the dominant technique used over the next several years; 89% of hematologists/oncologists are comfortable using IHC for treatment decisions but would welcome more supporting evidence on IHC-GEP concordance.

Equivocal pathology results and insufficient or inadequate specimens contribute to 9% of patients for whom a definitive COO classification could not be rendered (Figure 4B). Although IHC using the Hans algorithm is established and routine, it has not been standardized, with limited guidance on scoring and interpretation. Pathologists (including hematopathologists) report that weakly positive CD10 staining and focal MUM1/IRF4 staining are the most challenging scenarios. They favor clear, consensus guidelines for staining, scoring, and interpretation to standardize decision-making, reduce subjectivity, and lower equivocal and failure rates (Figure 7). Limited awareness of external quality control programs, such as UK NEQAS and NordiQC in Europe, in our interview set further support the need for stronger standardization efforts. Peer discussions and second opinions help resolve many equivocal results but delay reporting and require additional time. Reinforcing biopsy guidelines across the MDT may reduce insufficient and inadequate specimen issues.

While many hematologists/oncologists noted good readability and consistency, non-standardized hematopathology reports still left over 50% of hematologists/oncologists reporting interpretation difficulties or needing to follow up for missing data. Adopting a focused, disease-specific DLBCL template, rather than broad, impractical omnibus templates, following the footsteps of lung and breast cancers, would reduce variability and improve the clinical utility of key diagnostic, prognostic, and predictive stratification. Such a template should include the COO classification in the final DLBCL diagnosis and other features affecting treatment and prognosis (e.g., double-/triple-hit status). Although synoptic reporting is available for DLBCL, it is not broadly adopted, with only 25% of surveyed laboratory directors/pathologists (including hematopathologists) using a synoptic template that includes COO (Figure 5C). Synoptic reporting improves report completeness versus narrative formats [13,14] and has been associated with improved treatment rates and overall survival in some cancers [15]. Routine MDT meetings provide a venue to review results and clarify treatment decisions and have been shown to influence cancer diagnosis and management [16]. Together, these approaches could reduce burden on clinicians and increase the number of patients with prognostic COO classifications.

## 5. Conclusions

COO classification is guideline-recommended, widely adopted, and has been shown to be prognostic for patient outcomes, yet challenges remain. We found broad consensus among treating hematologists/oncologists that COO classification is a valuable component of DLBCL diagnosis and that COO is incorporated into treatment decision-making for most patients. In the US, COO classification is widely implemented, with most determinations performed by IHC for its accessibility, faster turnaround, lower cost, and favorable test reimbursement. Clinicians reported high confidence in IHC, citing high perceived concordance with GEP. Nonetheless, some gaps persist that can impede COO classifications or hinder its clinical utility: 6% of submitted specimens are unreportable due to tissue and quality limitations, 10–15% of samples initially have equivocal results (3% end unresolved), and the reporting templates used are non-standardized. Our findings support the need to develop consensus guidelines to standardize pathology reports and manage equivocal results alongside targeted education to reinforce the clinical implications of COO. Together, these actions can mitigate barriers and increase the share of patients with actionable COO classifications.

## Figures and Tables

**Table 1:** Hematologist/oncologist survey respondent demographics. Academic practice settings were defined as hospitals or centers affiliated with a university or medical school. Integrated community settings were defined as institutions that are part of a network or healthcare system. Community settings were defined as independently owned hospitals or practices that are not owned by a hospital or health system.

Hematologist/Oncologist Demographics (*n* = 60)		
**Cohort**	Segment	Percentage of Respondents
All Physicians	All	100%
Oncologist Specialty	Medical Oncologist	23%
	Hematological Oncologist	77%
Geographic Region	Northeast	23%
	Midwest	13%
	South	35%
	West	28%
Practice Setting	Academic Setting	35%
	Integrated Community Setting	35%
	Community Setting	30%
Years in Practice	2–5	12%
	6–15	32%
	16–25	35%
	26–40	22%
Annual DLBCL Patient Volume	<20	10%
	21–50	33%
	51–100	45%
	100+	12%

**Table 2:** Lab director/hematopathologist/pathologist survey respondent demographics. Academic labs were defined as labs affiliated with a university or medical school. Integrated community labs were defined as institutions that are part of a network or healthcare system. Community labs were defined as independently owned labs that are not owned by a hospital or health system. Commercial/reference labs were defined as independent for-profit labs providing centralized, high-volume testing for multiple institutions through send-out services; offering broad menus and standardized workflows; and typically not embedded in any single health system.

Lab Director/Pathologist Demographics (*n* = 60)		
**Cohort**	Segment	Percentage of Respondents
All Lab Directors and Pathologists	All	100%
Respondent Specialty	Lab Director	32%
	Pathologist	68%
Geographic Region	Northeast	13%
	Midwest	28%
	South	22%
	West	37%
Practice Setting	Academic Laboratory	38%
	Integrated Community Laboratory	35%
	Community Laboratory	20%
	Commercial/Reference Laboratory	7%
Pathology Board Certification	Board Certified in Hematopathology	44%
	Not Board Certified in Hematopathology	56%
Monthly DLBCL Sample Volume	<10	30%
	10–30	42%
	31–100	20%
	100+	8%

## Data Availability

The data underlying this article will be shared upon reasonable request to the corresponding author.

## Abbreviations

The following abbreviations are used in this manuscript:

ABC	Activated B-cell
BCL6	B-cell lymphoma 6
COO	Cell of origin
CD10	Cluster of differentiation 10
DLBCL	Diffuse large B-cell lymphoma
FF	Fresh frozen
FFPE	Formalin-fixed, paraffin-embedded
FISH	Fluorescence in situ hybridization
FNA	Fine-needle aspirate
GCB	Germinal center B-cell
GEP	Gene expression profiling
IHC	Immunohistochemistry
IRF4	Interferon regulatory factor 4
MDT	Multidisciplinary team
MUM1	Multiple myeloma oncogene 1
NGS	Next generation sequencing
NHL	Non-Hodgkin lymphoma
R-CHOP	Rituximab, cyclophosphamide, hydroxydaunomycin, vincristine sulfate (Oncovin), prednisone
TN	Treatment-naïve
